# Examining the impact of a social skills training program on preschoolers’ social behaviors: a cluster-randomized controlled trial in child care centers

**DOI:** 10.1186/s40359-020-00408-2

**Published:** 2020-04-23

**Authors:** Marie-Pier Larose, Isabelle Ouellet-Morin, Francis Vergunst, Frank Vitaro, Alain Girard, Richard E. Tremblay, Mara Brendgen, Sylvana M. Côté

**Affiliations:** 1 Université de Montréal, Montreal, Canada; 2 Université du Québec à Montréal, Montreal, Canada; 3 Université de Bordeaux, INSERM U1219, Bordeaux, France; 4 Centre de recherche du CHU Ste-Justine, Montréal, Canada

**Keywords:** Childcare-based intervention, Child development, Social skills, Early childhood education, Problem behavior

## Abstract

**Background:**

Preschoolers regularly display disruptive behaviors in child care settings because they have not yet developed the social skills necessary to interact prosocially with others. Disruptive behaviors interfere with daily routines and can lead to conflict with peers and educators. We investigated the impact of a social skills training program led by childcare educators on children’s social behaviors and tested whether the impact varied according to the child’s sex and family socio-economic status.

**Methods:**

Nineteen public Child Care Centers (CCC, *n* = 361 children) located in low socio-economic neighborhoods of Montreal, Canada, were randomized into one of two conditions: 1) intervention (*n =* 10 CCC; 185 children) or 2) wait list control (*n* = 9 CCC; 176 children). Educators rated children’s behaviors (i.e., disruptive and prosocial behaviors) before and after the intervention. Hierarchical linear mixed models were used to account for the nested structure of the data.

**Results:**

At pre-intervention, no differences in disruptive and prosocial behaviors were observed between the experimental conditions. At post-intervention, we found a significant sex by intervention interaction (*β intervention by sex* = − 1.19, *p* = 0.04) indicating that girls in the intervention condition exhibited lower levels of disruptive behaviors compared to girls in the control condition (*f2 effect size = −* 0.15). There was no effect of the intervention for boys.

**Conclusions:**

Girls may benefit more than boys from social skills training offered in the child care context. Studies with larger sample sizes and greater intervention intensity are needed to confirm the results.

**Trial registration:**

Current clinical trial number is ISRCTN84339956 (Retrospectively registered in March 2017). No amendment to initial protocol.

## Background

The use of early education and care services has substantially increased over the past four decades in most Western industrialized countries [1]. Early education and care services refer to regular group-based care of children prior to school entry (i.e., under age 5 years in North America) by someone other than the parents. Group-based child care centers (CCC) are one of the most important structured environments for early child socialization. Research suggests that exposure to high-quality child care in preschool settings has a positive effect on children’s social and cognitive school preparedness [2–4]. Benefits are particularly evident among children raised in poverty or in a low socio-economic status (SES) families [4–7]. Attending an early education and care setting is therefore an important preventive strategy for social adjustment and academic attainment problems [3, 8].

During the preschool years, children are more likely to exhibit disruptive behaviors such as aggression, non-compliance with rules and negative affectivity especially in social settings like CCCs [9]. This is because they are required to interact with many peers and educators for many hours each day and because they have not yet acquired sufficient self-control and the social skills necessary to communicate their needs and negative emotions [10, 11]. Emotional and cognitive immaturity in CCC settings may also be compounded by a phenomenon known as social contagion whereby preschoolers exposed to peers with disruptive behaviors mirror these behaviors or are forced to respond in similar ways in order to adapt to the social context (e.g. pushing, hitting, kicking) [12–14]. Children with disruptive behavioral problems tend to disrupt CCC daily routines, leading to conflict with peers and educators [15]. They are also more likely to be excluded from socially and cognitively stimulating activities and consequently to experience academic and social adjustment difficulties later on [15, 16]. It is therefore vital to provide child care environments that promote the development of good social relationships with peers and educators as early as possible so that children can enter the formal education system with adequate social and cognitive abilities [17].

### Children at higher risk of disruptive behavior problems

During the preschool years, boys and girls exhibit similar levels of disruptive behaviors, but males exhibit more problems after school entry [14, 18]. Studies show that early preventive interventions delivered in CCC settings can yield short- and long-term benefits [19–21]. However, the question of whether boys and girls respond differently to these interventions is not well-documented in the literature. Of five preschool intervention studies that targeted children’s socio-emotional development [22], only one reported testing the interaction between the experimental conditions and the children’s sex [23]. Girard and colleagues reported that an educator training intervention designed to scaffold peer interactions and use dramatic play reduced aggressive behaviors in boys but not girls [23]. This suggests that males and females may respond differently to disruptive behavioral intervention programs and further investigation of sex as a putative moderator is therefore warranted.

Another potentially important moderator of the effects of disruptive behavioral intervention programs is the SES of the child’s family. Children from low-SES families are more likely to exhibit disruptive behaviors from preschool to pre-adolescence when compared with children from higher SES families [14, 24]. Consequently, children from low-SES families are more prone to enter school with socio-emotional skills deficits that undermine school adjustment [15]. However, CCC attendance may counteract the influence of a socio-economically deprived family-environment on children’s socio-emotional skills by providing cognitive stimulation and socialization opportunities in a well-structured environment [25]. Children from low-SES families might therefore be more responsive to interventions delivered in CCC that target social-emotional skills development.

### Interventions on Children’s social development in child care context

Behavioral and cognitive management strategies in the context of preschools have shown positive short- and long-term effects on social behaviors, academic readiness and cognitive abilities, especially in the context of Head Start programs [20, 26–29]. However, outside of the Head Start literature, few studies have investigated the role of child care interventions on children’s socio-emotional development [22]. Doing so is important because the resources available to educators may vary between Head Start and community-based CCC settings. Head Start is a highly-structured government-run preschool program in which teachers have formal training in early childhood education and follow a prescribed curriculum focused on improving school readiness [30]. Community-based child care services, in contrast, may be run by public or private agencies, in which child care educators may not endorse a structured curriculum and may or may not have received formal training. Consequently, educators’ capacity to effectively implement social skills programs may vary widely between these contexts.

Previous CCC interventions have typically targeted caregiver-child relationship as their active ingredient and implemented a specific curriculum, i.e., activities around a certain theme [22]. One example is the Preschool Life Skills (PLS) which focuses on thirteen skills related to instruction-following, functional communication, delay tolerance, and friendship. Studies show that the PLS can significantly reduce disruptive behaviors in preschool children [21]. Additionally, educators reported that the social skills training was easy to incorporate into their daily routine and improved the social dynamics between children in their groups [21]. In this project, we evaluate a social skills training similar to the PLS – the “Minipally” program – which focuses on social skills development in a group context. The Minipally program is distinct that it is oriented less towards communication skills and preparedness for the school environment, and more towards social and emotional regulation skills.

### Objectives

Using a cluster-randomized controlled trial, we tested the impact of a social skills training program, delivered by child care educators, on children’s disruptive and prosocial behaviors. We also examined whether children’s sex and family SES moderated the impact of the program. We expected children exposed to the social skills training program to exhibit lower levels of disruptive behaviors and higher levels of prosocial behaviors at post-intervention compared to children in the control condition. Given the lack of evidence showing that children’s sex and family SES moderate the impact of social skills programs in CCC contexts, we did not have hypotheses about these variables.

## Methods

### Study design

**Fig. 1:**
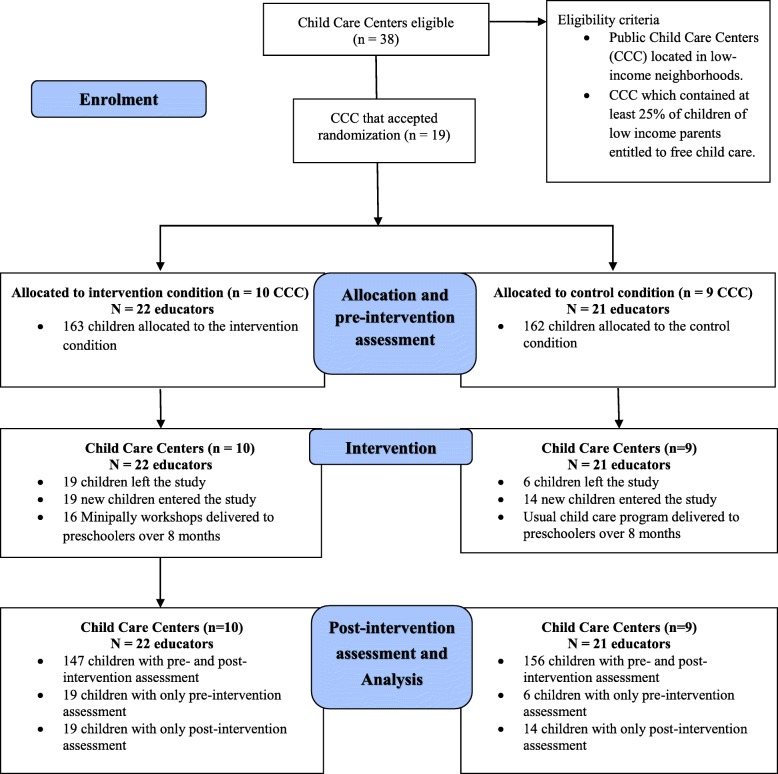
Minipally Trial Flow Chart. *Note.* CCC = Child Care Centers

Heads of 38 public CCC of the greater Montreal region were invited to participate in the study as they respected our eligibility criterion for participation: i.e., providing services to a minimum of 25% of children from low-income families and being in low-SES neighborhoods. Neighborhood SES was defined according to official provincial [31] and national criteria [32]. Lower-income families were those entitled to a special government subsidy program providing free child care access for families with an annual family income below CAN$20,000. After an information session, nineteen CCCs agreed to participate in the 8-month study. The CCCs were randomized with a 1:1 ratio to either: 1) the intervention condition (receiving the program in year 1) or 2) the wait list control condition (receiving the program in year 2) using a computer-generated randomization sequence. Each CCC included between one and 5 groups (mean = 2.32), *n =* 8 preschoolers led by an educator. Forty-three groups (*n =* 361 children) from 19 CCCs were recruited in September 2013 and took part in the study (Fig. 1: Trial Flow Diagram). Written consent to participate in the study were obtained from parents, educators and directors of the CCCs. The study was approved by the Sainte-Justine Hospital Ethical Research Committee (ref: 2014–565, 3738) and registered on a primary clinical trial registry prior to beginning data analysis. A detailed description of the study protocol describing the rationale behind the Minipally program and its evaluation was published shortly thereafter [33].

### Minipally curriculum

The Minipally program is an adaptation of an earlier social skills training programs for school-aged children – i.e. Fluppy program – which was developed by our research team and has shown long-term benefits for academic achievement, employment, income, delinquency and substance abuse [34, 35]. Over the past 20 years, experienced educational psychologists and psychoeducators have updated the Fluppy program to address the evolution of best practices in social skill training and adapt it to younger age groups, i.e. preschool-aged children. For example, in the school-aged program, children are taught how to deal with several emotions at the same time (e.g., feeling sad and upset) and to talk about their frustrations, while in the preschool version, children are taught to identify and name emotions and to manage their frustrations using age-appropriate stress-releasing techniques. Thus, while preschool-aged children are taught to use breathing techniques using the butterfly analogy, i.e. to breathe and raise their wings (arms) like a butterfly, school-aged children are taught to pause, withdraw from the situation if possible, and take five deep breaths.

The Minipally curriculum is delivered by each educator to her own group of children using a puppet via 16 play sessions over a period of 8 months. The puppet presents itself as a loyal and enthusiastic friend who visits the CCC to model prosocial behaviors and social inclusion by discussing/playing with his friends (other puppets) and with the children. The full curriculum includes generic components of social skills training programs: introduction to social contact (make and accept contact from others, make requests); problem solving (identifying the problem, generating solutions); self-regulation (deep breathing to calm down, accepting frustration, learning to share, tolerating frustration); and emotional regulation (identifying and expressing emotions, listening to the other). The skills taught in each workshop are presented in Table S1 in supplementary material.

Specifically, in each workshop, the educator calls on the Minipally puppet who then directly solicits the participation of each child and models adaptive social skills. Like children, Minipally feels great joys, but also has some difficulties with contact with others. The workshops are lively to solicit the participation and feedback of children as Minipally suggests ways for children to do things or asks them for suggestions. During the workshops, Minipally verbalizes a lot; he communicates everything he thinks and does in order to help children remember his actions, words, emotions and attitudes. Minipally is very attentive throughout the workshop as he congratulates children who exhibit the desired behaviors (i.e., wait for his turn, help another child) and encourages those who make efforts to practice the new skills presented. In other words, Minipally acts as a safe and friendly figure for children and a playful tool for child care educators to introduce new concepts and rules in a group context. Child care educators are also invited to reinvest the strategies presented by Minipally in natural settings on a day to day basis: they are encouraged to observe children during free play, reinforce positive behaviors as they occur and invite children to refer to what they learned during the last Minipally visit.

### Educator training and supervision

The program was implemented as follows. The 16 workshops of the Minipally curriculum were presented to the educators during a 2-day training delivered by trained professionals (i.e., psychoeducators). After the workshops the psychoeducators remained available by telephone for additional questions during the implementation of the curriculum by the educators. CCC directors were financially compensated for the replacement of the educators while they were trained. Next, the educators delivered the Minipally intervention over 8-months (one session every 2 weeks) and received 12 h (i.e., 4 × 3-h supervision; week 6, 12, 18 and 24 of the trial) of group supervision. During the supervision sessions, between 8 and 10 educators met with a psychoeducator to discuss the challenges associated with the implementation of the Minipally curriculum.

### Measures

#### Outcomes: disruptive and prosocial behaviors assessed by educators

Educators completed the Social Behavior Questionnaire [36] for each child in their group at pre- and post-intervention. Two dimensions of the questionnaire were used: a) *Disruptive Behaviors*, which included five opposition items (e.g., has been defiant or has refused to comply with an adult request), four impulsivity/hyperactivity items (e.g., has had difficulty waiting for his/her turn in games) and six physical aggression items (three reactive, e.g., has reacted aggressively when teased, and three non-reactive, e.g., has gotten into fights) (Cronbach alpha = 0.86); and b) *Prosocial Behaviors* (e.g., has helped other children, has shared his toys with others, has comforted a child who was upset; 7 items) (Cronbach alpha = 0.79). Educators rated each item using a 3-point Likert scale according to the frequency of the behavior in the last 2 weeks (0 = never, 1 = sometimes, and 2 = often). For each dimension, we created a cumulative score varying from 0 to 10, with 0 indicating that the child did not exhibit this behavior and 10 indicating that the child often exhibits this behavior.

### Covariates and moderators

#### Family sociodemographic characteristics

Before beginning the intervention, the child’s parents completed a questionnaire about their child’s CCC attendance details (e.g., number of hours per week, number of months since first attendance), the age and sex of their child, their family composition (e.g., number of siblings), and their socio-demographic background (education and income). A family SES score was then created by combining the maternal education and family income variables (i.e., total income in the household where the child lives most of the time). A low-SES score was assigned if the child lived in a household where the family earned less than CAN$20,000 per year and where the highest level of maternal education was a high school diploma. If the child was living in a household where the family was earning more than CAN$20,000, or where the mother had obtained any training following her high school diploma, the child was assigned to the middle-high SES group.

### Statistical analysis

#### Sample size calculation

Prior to the recruitment, we performed an a-priori power analysis to determine the sample size needed for the trial. The mean and standard deviation estimates for preschoolers’ disruptive and prosocial behaviours were taken from the Quebec Longitudinal Study of Children’s Development [24]. We did not have an estimate of the intra-class correlation (ICC) for CCC, so we estimated different scenarios using 0.1, 0.15 and 0.20 as the ICC coefficient and potential effect sizes (i.e., 0.3, 0.4 and 0.5) based on the difference in mean levels of disruptive and prosocial behaviours between the intervention and control conditions. We used Heo’s statistical procedure for cluster randomized trials with three-level units in our sample size estimation [37]. In other words, our calculation was based on the expected mean number of groups within each child care centers—i.e. 2 groups per child care center. Using the 0.15 ICC scenario, our power calculation indicated that 19 child care services would allow to detect a medium-size effect of the intervention on the selected outcomes, with 90% power at a 2-sided significance level of α = 5%. Our model can be stated as Y_ijk_ = β_0_ + δX_i_ + u_i_ + u_j (i)_ + e_ijk_; where Y_ijk_ is the post-intervention response of the i^th^ study participant in the j^th^ educator group nested in the k^th^ child care center, β_0_ represents the baseline value of our primary outcome, while δX_i_ is the main effect of the intervention (where X = 0 for wait list group and X = 1 for the intervention group), and the last three terms are random effects at every level of the trial analysis [37]. This scenario was chosen in accordance with our financial resources and the feasibility of the study [33]. The cluster randomization ensured that children from the control wait list condition were not exposed to the intervention. After completion of data collection, all control CCC received the social skills training.

### Preliminary analysis

#### Randomization balance analysis

Despite the use of a cluster randomization, there is still the possibility that individual characteristics are unequally distributed between the two experimental conditions. We therefore performed a series of preliminary analyses to compare the intervention and control conditions at baseline on a host of variables that may directly or indirectly impact the effect of the intervention (see Table 1). Only children’s age, the number of months of attendance at the CCC and family SES differed between the intervention and control groups. However, these variables were not significantly associated with any of the outcomes and were therefore not included as control variables based on the randomization balance analysis.

**Table 1:** Descriptive Statistics of the Sample According to Intervention Conditions

	Control	Intervention	*p-value*
	(*n* = 176)	(*n* = 185)	
Sex (*boy*) ^a^	86 (49.1%)	98 (52.7%)	0.57
Age *(months)*^b^	52.8 (5.0)	54.5 (4.5)	p < 0.01
	*164*	*165*	
Children with a developmental diagnosis ^a^	7 (4.3%)	10 (6.1%)	0.47
	*162*	*164*	
Siblings ^a^	128 (78.5%)	143 (86.7%)	0.12
	*163*	*165*	
Language spoken at home ^a^			0.56
French	123 (76.4%)	117 (71.3%)	
English	4 (2.5%)	4 (2.4%)	
Other	34 (21.1%)	43 (26.2%)	
	*161*	*164*	
Number of months the child attended childcare ^b^	39.4 (9.3)	40.2 (9.9)	0.46
	*151*	*159*	
Child care hours/week ^a^			0.26
Less than 30 h	24 (14.6%)	35 (21.2%)	
Between 30 and 40 h	104 (63.4%)	95 (57.6%)	
More than 40 h	36 (22.0%)	35 (21.2%)	
	*164*	*165*	
Family socio-economic status ^a^			0.04
Low socio-economic status	28 (18.7%)	15 (10.1%)	
Middle-high socio-economic status	122 (81.3%)	133 (89.9%)	
	*150*	*148*	

^a^ Frequency (%)

^b^ Mean (SD)

*Note1.* SD = Standard deviation

*Note 2.* We used bivariate analyses (t-test for continuous variables and chi-square for categorical variables) to verify whether socio-demographic characteristics of the child’s family were balanced between the intervention and control groups

#### Attrition analysis

No CCC withdrew from the study over the course of the intervention. However, 25 children left their CCC between pre- and post-intervention, representing a 7% attrition rate. These children were replaced by 33 newcomers (14 in the control condition and 19 in the intervention condition). If the new children entered their CCC in the first half of the trial (i.e., week 16 out of 32), they were included in the post-intervention assessment and in further analysis, after first obtaining parental consent. Children who entered the CCC after the 16th week of the intervention were not invited to participate in the study. In attrition analyses, we compared the 25 children who left the study with the 33 children who entered after the pre-intervention assessment (i.e., newcomers) and the 303 children who entered at pre-intervention and remained in the study. More children left the intervention condition than the control condition, but newcomers were equally distributed in both experimental conditions. There were no statistically significant differences between the children enrolled at baseline, those who left the study and those who entered later, in terms of sex, age and number of siblings. However, children who entered the intervention group later were more likely to come from middle-high SES families while children who entered the wait list group were more likely to come from low-SES families. We therefore controlled for family SES in all analyses.

#### Are there differences between experimental conditions at pre-intervention on children’s disruptive and prosocial behaviors?

We used hierarchical linear mixed models to examine differences in disruptive and prosocial behaviors between children in the intervention and control conditions at pre-intervention. No differences were found with respect to pre-intervention disruptive and prosocial behaviors (see Supplementary material Table S2). However, girls in the intervention group exhibited significantly higher levels of prosocial behaviors compared to girls in the control group and compared to boys from both the intervention group and the control group, respectively (*β intervention by sex* = 1.61, *p <* 0.01). We therefore controlled for pre-intervention levels of children’s prosocial behaviors in post-intervention models, in addition to assessing a potential moderating effect of children’s sex. For disruptive behavior, we did not find any significant interaction between the experimental condition and children’s sex, and consequently did not control for pre-intervention levels of disruptive behaviors in subsequent models.

### Main analysis

Hierarchical linear mixed models were used to estimate the main effects of the intervention on post-intervention disruptive and prosocial behaviors and to estimate if the impact of the intervention varied according to children’s sex and family SES. To account for variation in the number of children across CCCs, we used the restricted maximum likelihood estimator in every model. The analysis was performed in five steps.

First, because randomization was performed at the CCC level, we had to account for clustering in our data and we therefore ran an unconditional model to estimate the intra-class correlation (ICC) between clusters. The ICC is the proportion of variance in the outcome variable that is explained by the grouping structure of the hierarchical model [38]. It reports the amount of variation unexplained by any predictors in the model that can be attributed to the grouping variable, compared to the overall unexplained variance [38]. In the unconditional model, only the intercept was introduced as a fixed effect.

Second, we introduced the experimental condition variable as a main fixed predictor with and without the family SES covariate. Since the CCCs are the unit of randomization in this study, we expected variation between and within clusters and therefore accounted for this by introducing random effects. In other words, because children’s sex and family SES could vary within the same cluster, i.e., children from different SES backgrounds attended the same CCC, we introduced them as fixed and random effects for the adjusted and interaction models.

In subsequent models, we added an interaction term between our hypothesized moderators (i.e., children’s sex and family SES) and the experimental condition variable in the prediction of children’s disruptive and prosocial behaviors. Once again, the random effects specified in these models were the intercept, as well as family SES and children’s sex. Because of baseline differences between the experimental conditions found in preliminary analysis, we also added children’s pre-intervention prosocial behavior score as a fixed and random effect when assessing the moderating effect of children’s sex on the association between the experimental condition and post-intervention prosocial behavior.

Fourth, we performed pairwise comparisons between the intervention and the control group according to children’s sex and family SES, based on the mixed hierarchical model mean estimates. Finally, we estimated the effect sizes of the difference in means using the *f2* fixed effect size estimation [39] for hierarchical linear mixed models recommended by Lorah (2018) [40]. The *f2* effect size statistic represents the proportion of variance explained by the given fixed effects relative to the unexplained proportion of outcome variance. Effects of 0.02, 0.15 and 0.35 are considered small, medium and large respectively [41].

## Results

### Descriptive statistics

#### Participants

Children (*n* = 361) were distributed into 19 different CCCs. Table 1 shows that most children attended CCC for 30 to 40 h per week and that the number of boys and girls in the intervention group and the control group was roughly equal. Table 2 shows children’s raw scores for disruptive and prosocial behaviors at pre- and post-intervention according to the experimental conditions.

**Table 2:** Levels of Disruptive and Prosocial Behaviors by Intervention Conditions and Time of Assessment

	Control		Intervention	
Dependent variables	Pre-intervention	Post-intervention	Pre-intervention	Post-intervention
Disruptive behaviors ^a^	3.43 (0.20)	3.47 (0.28)	3.07 (0.20)	2.94 (0.27)
Prosocial behaviors ^a^	6.46 (0.24)	6.84 (0.22)	6.45 (0.24)	7.31 (0.22)

^a^ Mean (SD)

*Note.* SD = Standard deviation. Pre-intervention assessment was conducted in October and post-intervention assessment in June the following year

#### Implementation of Minipally

All educators were female, and most (85%) had a professional early education training. All educators in the intervention group received the two-day Minipally training. Implementation was monitored throughout the year via four half-day supervision sessions. At the last supervision session (week 24 out of 32 in the trial), all educators in the intervention group had implemented 12 of the 16 Minipally workshops. Thereafter, the exact number of workshops conducted by every educator was not monitored.

### Did the intervention have an impact on children’s social skills?

#### Disruptive behaviors

At post-intervention, the unconditional model showed that about 9% of the total variation in post-intervention disruptive behaviors was accounted for by differences between CCCs. When entering the experimental condition variable as a fixed effect, while adjusting for children’s family SES (*β* = 0.27, *p =* 0.52), we found no main effect of the intervention on children’s post-intervention disruptive behaviors (*β* = 0.39, *p =* 0.34). This suggested that the mean level of post-intervention disruptive behaviors was not different between the intervention and the control group. The ICC for this model dropped to 0.05, indicating that we accounted for a larger portion of the variation among the different CCCs and that less variation existed in the random intercepts of our model. Coefficients for the post-intervention models and their associated ICCs are presented in Table 3.

**Table 3:** Linear Mixed Models Linking Intervention Conditions to Disruptive and Prosocial Behaviors in Post-intervention

	Intervention					Intervention & Covariates				
	*β*	*SE*	*Df*	*p-value*	*ICC*	*β*	*SE*	*Df*	*p-value*	*ICC*
**Disruptive Behavior**										
**Unconditional model**										
**Random intercept**										
Intercept	3.19	0.2	17.55	< 0.01	0.09					
**Conditional Models**										
**Covariate & intervention variables**										
Intercept	2.94	0.27	17.43	< 0.01	0.08	2.99	0.29	18.52	< 0.01	0.05
Intervention	0.52	0.39	16.31	0.19		0.39	0.40	18.27	0.34	
Familial SES						0.27	0.42	98.93	0.52	
**Moderation models**										
***Children’s sex***										
Intercept						2.52	0.34	39.80	< 0.01	0.03
Intervention						0.98	0.46	36.72	0.04	
Familial SES						0.29	0.40	156.87	0.02	
Children’s sex						0.93	0.41	118.14	0.46	
Children’s sex * Intervention						−1.19	0.57	113.14	0.04	
***Familial SES***										
Intercept						3.01	0.30	17.86	< 0.01	0.05
Intervention						0.35	0.43	17.47	0.41	
Familial SES						0.17	0.67	146.49	0.80	
Familial SES * Intervention						0.17	0.86	110.96	0.84	
**Prososcial Behavior**										
**Unconditional model**										
**Random intercept**										
Intercept	7.08	0.16	14.95	< 0.01	0.08					
**Conditional Models**										
**Covariate & intervention variables**										
Intercept	7.31	0.22	14.52	< 0.01	0.07	7.28	0.23	17.76	< 0.01	0.03
Intervention	−0.47	0.31	13.44	0.16		−0.46	0.31	17.45	0.16	
Familial SES						−0.04	0.35	109.28	0.92	
**Moderation models**										
***Children’s sex***										
Intercept						4.22	0.46	167.29	< 0.01	< 0.01
Children’s pre-intervention level of prosocial behaviours						0.46	0.06	144.98	< 0.01	
Intervention						−0.17	0.37	251.65	0.65	
Familial SES						0.09	0.3	20.02	0.77	
Children’s sex						0.31	0.32	251.81	0.34	
Children’s sex * Intervention						−0.24	0.42	251.25	0.57	
***Familial SES***										
Intercept						7.34	0.23	18.19	< 0.01	0.03
Intervention						−0.48	0.34	17.72	0.17	
Familial SES						−0.06	0.45	110.91	0.89	
Familial SES * Intervention						−0.07	0.61	94.10	0.91	

*Note. SES* Socio-Economic Status, *B* Regression Coefficient, *SE* Standard Error, *Df* Degree of Freedom, *ICC* Intra-Class Correlation

#### Did child’s sex or the socio-economic status of the family moderate the impact of the intervention?

**Fig. 2:**
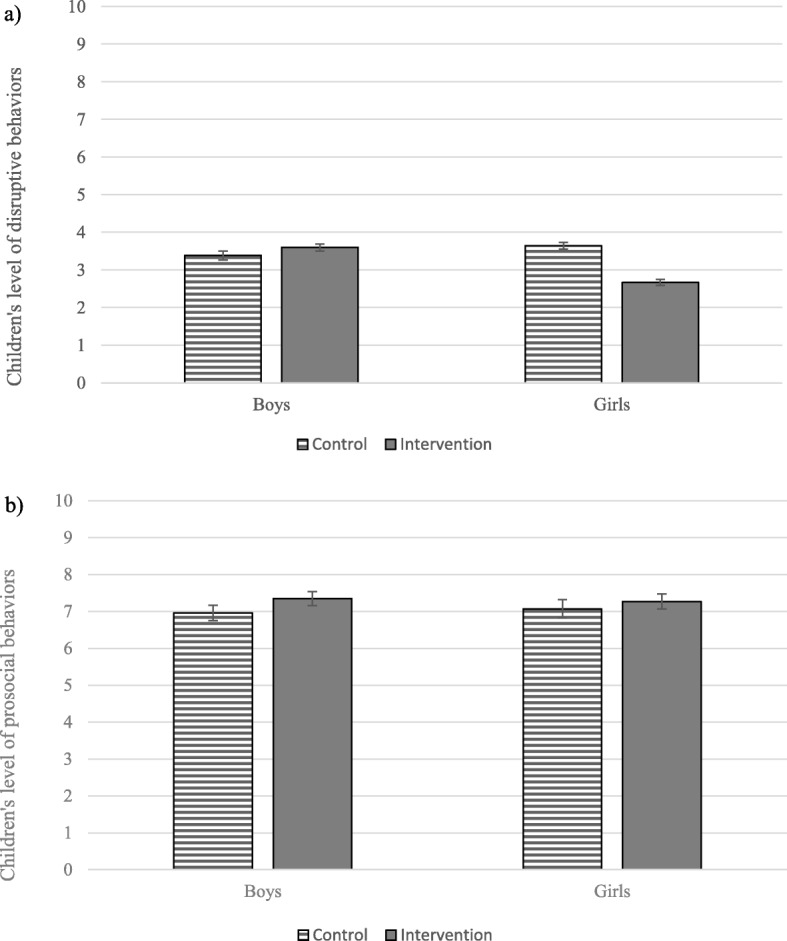
Children’s Levels of Disruptive (**a**) and Prosocial (**b**) Behavior in Post-intervention. *Note 1*. Mean score and 95% confidence intervals on children’s levels of disruptive (**a**) and prosocial (**b**) behavior in post-intervention according to intervention conditions and children’s sex. *Note 2.* Models adjusted for children’s family socio-economic status

We found a significant interaction between experimental conditions and children’s sex (*β* = − 1.19, *p =* 0.04, Fig. 2a), indicating lower levels of post-intervention disruptive behaviors in the intervention group compared to the control group for girls (*F =* 4.19, *df =* 43.08*, p =* 0.04; *f2 effect size = −* 0.15). For boys, there was no difference in post-intervention disruptive behaviors between the intervention group and the control group (*F =* 0.37, *df =* 49.20, *p* = 0.55; *f2 effect size =* 0.04).

We also investigated the potential moderating effect of family SES, but no significant interaction was found (*β* = 0.17, *p =* 0.86; *f2 effect size for middle-high SES* children < 0.01, *f2 effect size low SES* <  0.01).

#### Prosocial behaviors

For prosocial behaviors, there was no main effect of the intervention and no moderation effect of children’s sex or family SES. Coefficients and ICCs for all tested models are presented in Table 3. Figure 2b shows the prosocial scores according to experimental conditions and children’s sex.

#### Sensitivity analysis

We performed the same set of analyses with a restricted sample of children who had both pre- and post-intervention assessments (i.e., newcomers were excluded from the sensitivity analysis). We found the same patterns of results, namely that the intervention led to a decrease in disruptive behaviors among girls only but had no impact on prosocial behaviors for girls or boys.

## Discussion

This study used a cluster-randomized controlled trial design to test the impact of a social skills training program on children’s social behaviors in Child Care Centers in low-SES neighborhoods. Using hierarchical linear mixed models, we found that the sex of the child moderated the impact of the social skills training program, reducing the level of disruptive behaviors for girls but not for boys. The failure to find an effect for prosocial behaviors may be due to the high levels of prosocial behaviors in the experimental conditions at pre-intervention, leaving little room for improvement (i.e., ceiling effects). Furthermore, we found no evidence that the SES of the child’s family moderated the impact of the intervention.

### Examination of the evaluated intervention

With respect to disruptive behaviors, our results are consistent with earlier findings from a similar social skills intervention developed by our research team for school-aged children– the “Fluppy program” [42] – which found that disruptive behaviors at the end of the 8-month intervention were reduced for girls but not for boys [42]. One explanation for the observed sex differences is the highly verbal nature of these interventions. Sex differences in children’s verbal abilities are well-documented, particularly early in development [43, 44], so it is possible that the content and delivery of the interventions were not sufficiently accessible to boys. Indeed, the Minipally and Fluppy programs are specifically designed to improve social skills that frequently depend on verbal skills such as the ability to articulate questions or describe emotions.

Thus, while girls might be receptive to educator-led workshops that focus on enhancing social skills and reducing disruptive behaviors, this might not be the best approach for boys, who might instead benefit from educator-led dramatic play sessions, stronger educator-child relationships, and supervised peer play to scaffold social competences [23, 45, 46]. More broadly, our results corroborate the hypothesis that children’s sex is an important moderator of the impact of a social skills training program during early childhood and possibly later.

A further consideration for future studies is that adding a parenting component to the Minipally program could increase its impact. According to a recent meta-analysis, interventions with a parent component, either alone or in combination with other components, are more likely to benefit children who exhibit high levels of behavioral problems [47]. Future studies should therefore examine the unique and combined impact of child care-based and parenting-based interventions on children’s social behaviors when designing new interventions and early childhood politics.

Finally, previous work shows that social skills training programs for childhood disruptive behaviors are effective only if they are of moderate-to-high intensity [47]. It is possible that our intervention lacked the intensity necessary to significantly increase children’s prosocial behaviors and reduce disruptive behaviors in boys. The educators in our trial conducted at least 12 out of 16 workshops in the Minipally child curriculum, but their reinvestment activities (i.e., follow-up activities throughout the week) were not monitored. A higher intensity intervention with systematic reinvestment activities would arguably have had a greater impact on children’s social skills, especially for those exposed to risk factors in their home environment.

### Strengths and limitations

The strengths of this study are its cluster-randomized experimental design, low level of cluster (0%) and individual attrition (7%), and the use of hierarchical linear mixed models, which accounted for the nested structure of randomization. The study had good ecological validity. It was implemented in community-based CCCs by educators who, apart from receiving a 2-day training and 12 h of supervision for the social skills program, had only a two-year professional degree (after high school) in early childhood and child care education.

The study has several limitations. First, we underestimated the ICC of the data in our sample size calculation, which, when combined with our modest sample size, limited our capacity to detect small effects. Future studies should replicate the intervention using larger samples and test a putative interaction with children’s sex and family SES, as well as other potential moderators, such as children’s baseline levels of prosocial and disruptive behaviors. Second, children’s behavioral questionnaires were completed by the educator who also delivered the Minipally program. Childcare educators are a reliable source of information on disruptive behaviors because of their established ability to distinguish between normative and atypical behaviors[48, 49]; However, since the educators were involved in both the implementation of the intervention and the pre- and post-intervention behavioral assessments, this may have introduced a bias. For instance, due to their proximity to the project, educators in the intervention group may have noticed smaller improvement in children’s behaviors than educators in the control group. Nevertheless, it is unlikely that such bias would explain the different impact of the intervention on disruptive behaviors between boys and girls. The decision to rely on the CCC educators who participated in the study was based on extensive literature that shows there is only weak to moderate agreement in social skills evaluations between raters [50]. Social skills are highly context specific, and the skills necessary to function at home are considerably different from those required in group contexts typical of CCC settings [50]. Future studies seeking to replicate our intervention should consider evaluating children’s social competences based on assessments performed by independent raters. The use of objective tests – for example “The white crayon does not work …” task by Ostrov et al. [51] in which children are asked to participate in a group drawing exercise – should be considered in future studies to examine the impact of a social skills training program on children’s social behaviors. Also, a follow-up assessment at school entry with kindergarten teachers who have not been involved in the project may yield more reliable results. Finally, we did not track the number of workshops implemented by child care educators – we only know that all educators performed 12 or more of the 16 workshops during the implementation year. Future studies should include a comprehensive implementation and content validity evaluation.

## Conclusions

CCCs provide one of the earliest opportunities to equip children with social skills that will benefit them for the rest of their lives [52]. This study adds to a small but growing body of literature suggesting there may be important sex differences in children’s responsiveness to early psychosocial interventions. Preschool programs that provide social skills training with higher intensity, a defined educative curriculum, and parent engagement may help reduce behavior problems and enhance social skills with long-term benefits to individuals and society.

## Supplementary information

**Additional file 1: Table S1.** Skills taught in the Minipally program by workshops.

**Additional file 2: Table S2.** Linear Mixed Models Linking Intervention Conditions to Disruptive and Prosocial Behaviors in Pre-intervention.

## Data Availability

The datasets generated and/or analyzed during the current study are not expected to be available in accordance with the ethical approval received from the Ethical Research Committee: CHU Saint-Justine for confidentiality reasons.

## Abbreviations

CCC: Child care centers
ICC: Intra-class correlation
PLS: Preschool life skills
SES: Socio-economic status
