# Supplementary material for: Examining the impact of a social skills training program on preschoolers’ social behaviors: a cluster-randomized controlled trial in child care centers
Source: BMC Psychol. 2020 Apr 23;8:39. doi: 10.1186/s40359-020-00408-2 (PMC7181512; doi:10.1186/s40359-020-00408-2)
Supplement: Supplementary file 1 — Additional file 1: Table S1. Skills taught in the Minipally program by workshops. [file 40359_2020_408_MOESM1_ESM.docx]

# **Supplementary material: Table S1**

# Table S1: Skills taught in the Minipally program by workshops.

| Theme 1: Introduction to social contact |
| --- |
| 1. Initiating contact |
| 1. Making request |
| 1. Accepting others |
| Theme 2: Emotional regulation |
| 1. Being happy |
| 1. Being sad |
| 1. Being upset |
| 1. Recognize our own emotions |
| 1. Listening to others’ emotions |
| Theme 3: Self-control |
| 1. Waiting for his turn |
| 1. Listen to others |
| 1. Stop action to calm himself |
| 1. How to control his frustration |
| Theme 4: Problem solving |
| 1. Breath to calm himself |
| 1. Learn how to share |
| 1. Facing sadness |
| 1. Facing frustration from others |
