# Supplementary material for: Examining the impact of a social skills training program on preschoolers’ social behaviors: a cluster-randomized controlled trial in child care centers
Source: BMC Psychol. 2020 Apr 23;8:39. doi: 10.1186/s40359-020-00408-2 (PMC7181512; doi:10.1186/s40359-020-00408-2)
Supplement: Supplementary file 2 — Additional file 2: Table S2. Linear Mixed Models Linking Intervention Conditions to Disruptive and Prosocial Behaviors in Pre-intervention. [file 40359_2020_408_MOESM2_ESM.docx]

**Supplementary material Table S2**

Table S2: Linear Mixed Models Linking Intervention Conditions to Disruptive and Prosocial Behaviors in Pre-intervention

| **Disruptive Behavior** | | | | | | | | | | |
| --- | --- | --- | --- | --- | --- | --- | --- | --- | --- | --- |
| **Unconditional model** | | | | | | | | | | |
| **Random intercept** | **Intervention** | | | | | **Intervention & Covariates** | | | | |
|  | *β* | *SE* | *Df* | *p-value* | *ICC* | *β* | *SE* | *Df* | *p-value* | *ICC* |
| Intercept | 3.25 | 0.15 | 18.19 | < 0.01 | 0.03 |  |  |  |  |  |
| **Conditional Models** | | | | | | | | | | |
| **Covariate & intervention variables** | **Intervention** | | | | | **Intervention & Covariates** | | | | |
|  | *β* | *SE* | *Df* | *p-value* | *ICC* | *β* | *SE* | *Df* | *p-value* | *ICC* |
| Intercept | 3.07 | 0.2 | 15.71 | < 0.01 | 0.02 | 3.08 | 0.21 | 14.84 | < 0.01 | 0.01 |
| Intervention | 0.36 | 0.29 | 15.25 | 0.23 |  | 0.19 | 0.28 | 15.48 | 0.51 |  |
| Familial SES |  |  |  |  |  | 0.53 | 0.39 | 179.09 | 0.18 |  |
| **Moderation models** | **Intervention** | | | | | **Intervention & Covariates** | | | | |
| ***Children's sex*** | *β* | *SE* | *Df* | *p-value* | *ICC* | *β* | *SE* | *Df* | *p-value* | *ICC* |
| Intercept |  |  |  |  |  | 2.77 | 0.29 | 29.9 | < 0.01 | < 0.01 |
| Intervention |  |  |  |  |  | 0.6 | 0.4 | 29.93 | 0.14 |  |
| Familial SES |  |  |  |  |  | 0.54 | 0.39 | 192.87 | 0.17 |  |
| Children's sex |  |  |  |  |  | 0.63 | 0.38 | 113.79 | 0.10 |  |
| Children's sex * Intervention |  |  |  |  |  | -0.82 | 0.53 | 119.16 | 0.13 |  |
|  | **Intervention** | | | | | **Intervention & Covariates** | | | | |
| ***Familial SES*** | *β* | *SE* | *Df* | *p-value* | *ICC* | *β* | *SE* | *Df* | *p-value* | *ICC* |
| Intercept |  |  |  |  |  | 3.04 | 0.22 | 13.71 | < 0.01 | 0.01 |
| Intervention |  |  |  |  |  | 0.23 | 0.31 | 14.13 | 0.47 |  |
| Familial SES |  |  |  |  |  | 0.26 | 0.49 | 134.18 | 0.60 |  |
| Familial SES * Intervention |  |  |  |  |  | 0.12 | 0.66 | 126.51 | 0.86 |  |
| **Prososcial Behavior** | | | | | | | | | | |
| **Unconditional model** | | | | | | | | | | |
| **Random intercept** | **Intervention** | | | | | **Intervention & Covariates** | | | | |
|  | *β* | *SE* | *Df* | *p-value* | *ICC* | *β* | *SE* | *Df* | *p-value* | *ICC* |
| Intercept | 6.46 | 0.16 | 14.82 | < 0.01 | 0.06 |  |  |  |  |  |
| **Conditional Models** | | | | | | | | | | |
| **Covariate & intervention variables** | **Intervention** | | | | | **Intervention & Covariates** | | | | |
|  | *β* | *SE* | *Df* | *p-value* | *ICC* | *β* | *SE* | *Df* | *p-value* | *ICC* |
| Intercept | 6.45 | 0.24 | 14.77 | < 0.01 | 0.06 | 6.48 | 0.23 | 16.39 | < 0.01 | 0.03 |
| Intervention | 0.01 | 0.34 | 14.09 | 0.98 |  | -0.03 | 0.31 | 16.39 | 0.92 |  |
| Familial SES |  |  |  |  |  | -0.21 | 0.39 | 144.54 | 0.59 |  |
| **Moderation models** | **Intervention** | | | | | **Intervention & Covariates** | | | | |
| ***Children's sex*** | *β* | *SE* | *Df* | *p-value* | *ICC* | *β* | *SE* | *Df* | *p-value* | *ICC* |
| Intercept |  |  |  |  |  | 6.93 | 0.28 | 34.92 | < 0.01 | 0.02 |
| Intervention |  |  |  |  |  | -0.82 | 0.39 | 34.66 | 0.04 |  |
| Familial SES |  |  |  |  |  | -0.28 | 0.38 | 191.07 | 0.46 |  |
| Children's sex |  |  |  |  |  | -0.88 | 0.36 | 116.8 | 0.02 |  |
| Children's sex * Intervention |  |  |  |  |  | 1.61 | 0.51 | 122.76 | < 0.01 |  |
|  | **Intervention** | | | | | **Intervention & Covariates** | | | | |
| ***Familial SES*** | *β* | *SE* | *Df* | *p-value* | *ICC* | *β* | *SE* | *Df* | *p-value* | *ICC* |
| Intercept |  |  |  |  |  | 6.54 | 0.24 | 16.42 | < 0.01 | 0.03 |
| Intervention |  |  |  |  |  | -0.23 | 0.33 | 16.44 | 0.51 |  |
| Familial SES |  |  |  |  |  | -0.21 | 0.47 | 111.24 | 0.65 |  |
| Familial SES * Intervention |  |  |  |  |  | 0.62 | 0.65 | 107.71 | 0.35 |  |

*Note.* SES= Socio-Economic Status, B= Regression Coefficient, SE= Standard Error, Df= Degree of Freedom, ICC= Intra-Class Correlation
